# Effects of macronutrient intake on fuel utilization: potential sex differences

**DOI:** 10.1186/1550-2783-12-S1-P39

**Published:** 2015-09-21

**Authors:** Meredith G Mock, Katie R Hirsch, Erica J Roelofs, Eric T Trexler, Abbie E Smith-Ryan

**Affiliations:** 1 Applied Physiology Laboratory, Department of Exercise and Sport Science, University of North Carolina at Chapel Hill, Chapel Hill, NC 27599, USA

## Background

Evidence suggests that women oxidize more fat for fuel at rest than males. Potential sex differences in fuel utilization during exercise remain unclear. Alterations in diet may influence substrate utilization by altering substrate availability and metabolic enzyme activity. Reduced carbohydrate (CHO) intake has been shown to lower respiratory exchange ratio (RER) over time, which may improve aerobic endurance. The purpose of this study was to explore potential sex differences in the relationship between habitual macronutrient distribution and substrate utilization during exercise.

## Methods

Twenty-eight recreationally active college-aged participants (12 females, 16 males; mean ± SD; Age = 22.7 ± 4.1 yrs, BMI = 23.3 ± 2.7 kg·m^2^) completed a three-day food log. Participants were provided with detailed instructions for accurately logging food intake and portion sizes. Logs were analyzed using The Food Processor software (ESHA Research, Salem, OR, USA) for total calories, estimated energy requirements (EER), CHO (g/kg), fat (g/kg); and protein (PRO; g/kg). RER was analyzed via indirect calorimetry (Parvomedics TrueOne 2400) during a maximal oxygen consumption (VO_2 _max) test to exhaustion on a cycle ergometer and during a submaximal, six-minute cycling test. The six-minute cycling test was completed at a workload between 60% of ventilatory threshold and VO_2 _max. RER was collected throughout and averaged every minute.

## Results

For men, there was a significant positive correlation between CHO and RER at both 1 min (p = 0.012; R = 0.613) and 3 min (p = 0.013; R = 0.608) of high-intensity exercise, with no significant relationships with PRO or FAT. For women, there was a significant positive correlation with CHO and RER at 2 min (p = 0.008; R = 0.724); as well as a significant correlation between PRO and RER at 3 min (p = 0.010; R = 0.708). During high-intensity exercise, women demonstrated a significantly higher RER (p = 0.016) compared to men. Macronutrient intake analysis revealed a significant positive correlation between PRO and FAT (p = 0.007; R = 0.496) in both groups. For men, both CHO and PRO positively correlated with FAT (p = 0.040; R = 0.518; p = 0.010; R = 0.623) but only PRO and FAT were correlated in women (p = 0.009, R = 0.712). When energy intakes were below EER in the total group, PRO and FAT demonstrated a positive relationship.

## Conclusions

Contrary to the expected relationship, a positive correlation between RER and PRO intake was seen in women, with no relationship to fat intake. In contrast, higher CHO intake resulted in higher RER for men. Future studies should evaluate long-term effects of dietary changes on exercise fuel utilization. The ability to maximize fat utilization during exercise may be beneficial for longer aerobic events, as well as for weight loss. CHO and PRO correlated with FAT intake in men, but only PRO and FAT correlated in women, possibly suggesting women who consciously consume higher relative amounts of protein are aware of the health benefits of dietary fat.

